# Fused Deposition Modeling of Microfluidic Chips in Transparent Polystyrene

**DOI:** 10.3390/mi12111348

**Published:** 2021-10-31

**Authors:** Markus Mader, Christof Rein, Eveline Konrat, Sophia Lena Meermeyer, Cornelia Lee-Thedieck, Frederik Kotz-Helmer, Bastian E. Rapp

**Affiliations:** 1 Laboratory of Process Technology, NeptunLab, Department of Microsystems Engineering (IMTEK), University of Freiburg, 79110 Freiburg im Breisgau, Germany; Markus.Mader@neptunlab.org (M.M.); Christof.Rein@Neptunlab.org (C.R.); Eveline.Konrat@Neptunlab.org (E.K.); Bastian.Rapp@Neptunlab.org (B.E.R.); 2 Institute of Cell Biology and Biophysics, Department of Cell Biology, University of Hannover, 30419 Hannover, Germany; Meermeyer@Cell.Uni-Hannover.de (S.L.M.); lee-thedieck@cell.uni-hannover.de (C.L.-T.); 3 Freiburg Materials Research Center (FMF), University of Freiburg, 79104 Freiburg im Breisgau, Germany; 4 FIT Freiburg Center of Interactive Materials and Bioinspired Technologies, University of Freiburg, 79110 Freiburg im Breisgau, Germany

**Keywords:** 3D printing, additive manufacturing, fused deposition modeling, microfluidics, polystyrene, cell cultures

## Abstract

Polystyrene (PS) is one of the most commonly used thermoplastic materials worldwide and plays a ubiquitous role in today’s biomedical and life science industry and research. The main advantage of PS lies in its facile processability, its excellent optical and mechanical properties, as well as its biocompatibility. However, PS is only rarely used in microfluidic prototyping, since the structuring of PS is mainly performed using industrial-scale replication processes. So far, microfluidic chips in PS have not been accessible to rapid prototyping via 3D printing. In this work, we present, for the first time, 3D printing of transparent PS using fused deposition modeling (FDM). We present FDM printing of transparent PS microfluidic channels with dimensions as small as 300 µm and a high transparency in the region of interest. Furthermore, we demonstrate the fabrication of functional chips such as Tesla-mixer and mixer cascades. Cell culture experiments showed a high cell viability during seven days of culturing, as well as enabling cell adhesion and proliferation. With the aid of this new PS prototyping method, the development of future biomedical microfluidic chips will be significantly accelerated, as it enables using PS from the early academic prototyping all the way to industrial-scale mass replication.

## 1. Introduction

Microfluidics is a growing technology for controlling and alternating the behaviour of fluids at the sub-millimetre length scale. It offers distinct advantages for analytic applications in chemistry and biology, such as a reduction in material consumption, simplified fluid-dynamics as well as processing and screening of individual cells [1,2]. Furthermore, microfluidic devices can incorporate additional functionalities such as enzymatic processing, micropumps and microvalves or incubation controls [3,4,5,6]. Recently, microfluidics found a broad range of applications in drug discovery [7], gene sequencing [8], proteomics [9], diagnostics [10], tissue engineering [11] and organ-on-chip [12].

Early microfluidic chips were fabricated in glass or silicon, which requires etching processes limiting the chip’s geometry to mostly 2.5-dimensional shapes. Furthermore, complex cleanroom protocols are required for chip fabrication as, e.g., chip development in glass is a slow and expensive process [13]. Since the introduction of soft lithography, replication using polydimethylsiloxane (PDMS) became the gold standard for prototyping of microfluidic chips due to the material’s low price, transparency, gas permeability and ease of manufacturing [14,15,16]. However, there are several drawbacks of soft replication for microfluidic applications, such as the need for a replication master and the fact that additional bonding steps are required for sealing of the replication chips. Furthermore, there is no possibility of arbitrary 3D fabrication, as PDMS casting is limited to 2.5-dimensional structures [15]. 

In consequence, additive manufacturing (AM) recently gained significant attention for the fabrication of microfluidic chips, as it allows for true three-dimensional arbitrary designs without the need for additional bonding thus enabling intricate and complex channel systems [17]. Furthermore, the chip fabrication is significantly simplified as the device is constructed by computer aided design (CAD), making AM ideal for prototype generation [18,19]. One very popular AM technique is fused deposition modelling (FDM), where a thermoplast is extruded via a heated nozzle to sequentially build up three-dimensional objects layer-by-layer. FDM is an interesting technique for microfluidic prototyping as it is the ideal AM technology for thermoplast processing at reasonable low instrumentation invests [20,21,22]. In principle, FDM enables a smooth transition of lab-scale devices to the industry scale, as the same materials can be used for both chip development and later mass-production, most likely using high-throughput replication techniques such as injection moulding (IM). Several microfluidic devices have already been realized using FDM, such as microfluidic mixers and even sub-100 µm structures have been shown [23,24]. Unfortunately, the materials which have been processed using FDM for the fabrication of microfluidic systems have been limited to polylactic acid (PLA) [25,26], acrylonitrile butadiene styrene (ABS) [27], thermoplastic polyurethan (TPU) [24] and polymethylmethacrylate (PMMA) [17]. 

Until now, one of the most important thermoplastic materials is missing: polystyrene (PS). PS is one of the most commonly used materials in biology and cell culture for Petri dishes, chamber slide systems, multi-well plates and culture flasks, due to its low cost and excellent material properties such as high optical transparency and biocompatibility [28,29,30]. PS would also be an ideal material for microfluidic life science applications. Unfortunately, at the laboratory scale, the shaping of microstructures is demanding and has limited the usage of PS, especially in rapid prototyping [31,32]. On the industrial scale, PS can be easily processed using fully automated manufacturing techniques such as IM which allow for inexpensive structuring at high-throughput. Nevertheless, IM has the inherent problem of being an extremely inflexible process due to the high moulding tool cost [33]. On the laboratory scale, several microstructuring methods for PS prototyping have been reported, including hot embossing against epoxy or PDMS moulds. Our lab recently introduced liquid photocurable PS prepolymers, consisting of PS dissolved in styrene which can be cured and replicated using soft PDMS templates via photopolymerization [34]. However, these methods require the time-consuming generation of a mould beforehand [35,36,37]. Subtractive laser structuring and micromilling of PS have been reported to be flexible methods for the fabrication of open microfluidic channel structures [38,39]. A variation of micromilling termed “shrinky-dink” microfluidics has been introduced, where biaxial stretched PS foils are first structured and subsequently shrunken to the microscale in a heat treatment [40,41]. Nevertheless, all of these prototyping methods only allow the generation of 2.5-dimensional open microfluidic structures, which require subsequent bonding. In consequence, there is a high demand for PS prototyping with FDM technology in order to overcome these limitations.

In this work, we present, for the very first time, FDM 3D printing of transparent microfluidic devices in PS. We optimized the printing process, allowing the fabrication of microfluidic channel structures with a minimum feature size of 300 µm and a high transparency in the region of interest. The FDM printing process allows the fabrication of fully functional embedded microfluidics within 1 h, demonstrating that an industrially relevant thermoplastic polymer material can be structured fast and efficiently using established 3D printing technologies. By allowing a facile fabrication of complex PS microfluidic prototypes, it will be possible to accelerate the development of upcoming microfluidic devices and prototypes for biomedical applications using one of the most relevant thermoplastic materials for the mass-market.

## 2. Materials and Methods

### 2.1. Printing Materials 

PS granulates (avg. M_w_ = 350,000, avg. M_n_ = 170,000), ethanol absolute, Dulbecco’s phosphate buffered saline (DPBS) with CaCl_2_ and MgCl_2_ (DPBS (+/+)), DPBS without CaCl_2_ and MgCl_2_ (DPBS (−/−)), Triton-X100, propidium iodide (PI), Hoechst 33342, foetal bovine serum (FBS), calcein acetoxymethyl (calcein-AM), penicillin-streptomycin (P/S) and RPMI-1640 Medium with L-glutamate and sodium bicarbonate were purchased from Sigma-Aldrich (Taufkirchen, Germany). Disposable PS dishes, tissue culture poly styrene (TCPS) 24-well plates and 75 cm^2^ cell culture flasks were purchased from Greiner Bio-One GmbH (Frickenhausen, Germany). Mouse L-929 fibroblasts (NCTC Clone 929) were purchased from DSMZ (Braunschweig, Germany). Replisil 22 N two component silicon was purchased from Siltecs (Weiler-Simmerberg, Germany).

### 2.2. Filament Preparation

A continuous PS filament was prepared by extruding the PS granulates using a twin-screw extruder (Teach-Line ZK 25T, Collin, Germany) at a temperature of 220 °C with a material throughput of 1.5 kg/h. A nozzle with a diameter of 2.85 mm was used and the extruded PS filament was immediately cooled in a water bath. The extruded filament was pulled with a constant speed using a granulator unit of type M50/80 (Hellweg, Germany) without a blade to obtain a continuous filament with a mean diameter of about 2.6 mm. 

### 2.3. Fused Deposition Modelling

All FDM printing models were designed using Autodesk Inventor Professional 2019 and exported as STL-files. The STL-files were sliced using the slicing software Ultimaker Cura 4.7.1. FDM printing of transparent PS was performed using a commercial FDM printer (Ultimaker 2+, Ultimaker B.V., Utrecht, The Netherlands) equipped with a 0.4 mm nozzle. The slicing and printing parameters were optimized to print transparent microfluidic devices. A filament diameter of 2.6 mm was set in the slicing software. Parts were printed with a layer thickness of either 100 or 50 µm. A line width of 0.35 µm and a printing speed of 25 mm/s was used. It was found that a print head temperature of 240 °C without additional fan cooling delivered the best printing results. Good adhesion to the print bed was achieved with a print bed temperature of 70 °C. To reduce warping of the bigger parts, the print bed was treated with 3DLAC spray (3DLAC, Zamora, Spain). To print PS with higher transparency, we increased the flow rate for the bottom layer, top layer and infills to 120% and set the infill-wall overlap to 10% to reduce air inclusion. Infill was set to 100% and was printed as a line grid with every other layer perpendicular to the layer before. The flow rate for wall structures was set to 100% for higher accuracy. For the bridging structures during the printing of embedded microfluidic channels, the bridge flow rate was set to 60% to prevent sagging. In addition, the cooling fan was turned on during the printing of a bridge structure to further decrease sagging. 

To evaluate possible channel cross-section geometries and printing accuracy of embedded and open channels compared to the original CAD design, a series of straight test channels were printed with a channel size of 600 µm for the geometry analysis and 1000-200 µm for analysis of the printing accuracy. The FDM-printed PS parts were broken at different positions to examine and analyse the channel dimensions using a light microscope (VHX 6000, Keyence Corporation, Osaka, Japan).

To improve channel transparency, we printed open channel structures directly onto commercial PS substrates with a thickness of 1.1 mm. This strategy was previously reported for channel fabrication in PLA [25]. The PS substrate was fixed on the print bed before the 3D print was started. The print bed level was manually adjusted to take the substrate thickness into account. The deposited PS strands bond to the PS substrate upon deposition, forming closed and leakproof channels. 

### 2.4. Microfluidic Experiments

To proof the function of the FDM-printed microfluidic devices, the channels were filled with coloured water using syringes. This was either done manually or automatically using a syringe pump (Legato 210, KDScientific, Holliston, MA, USA). For using the syringe pump, dispensing needles (1”, Vieweg, Kranzberg, Germany) were plugged onto a FDM-printed tubing connector, fixed with epoxy glue and connected to the syringes via tubes. The coloured water was pumped through the microfluidic channels with pumping rates up to 10 mL/min.

The mixing efficiency was determined according to a procedure described in the literature, where the mixing efficiency is calculated from a photograph based on the standard deviation of normalized pixel intensity of coloured liquid inside the microfluidic channel [42]. For the determination of mixing efficiency *c_m_*, a photograph of the microfluidic channel filled with dyed liquids was first converted to greyscale using the software CorelDRAW. Afterwards a small region within the channel was selected, cut and analysed using the histogram setting of CorelDRAW to determine the mean pixel intensity *I_m_* as well as the standard deviation *σ*. Each selected region for intensity analysis contained at least 50 pixels. Three different regions were taken from the microfluidic channels after the respective mixing features. Mixing efficiency was calculated using the following equation, with higher *c_m_* indicating better mixing efficiencies.

*c_m_* = 1 − *σ*/*I_m_* (1)

### 2.5. Contact Angle Measurements

Contact angles were measured using an OCA 15 Pro (Dataphysics, Rock Hill, SC, USA). The surfaces were cleaned with ethanol before measuring. Droplets of water (5 µL) were applied to the surface and the static contact angle was measured.

### 2.6. UV/Vis Measurements

The UV/Vis spectrometer Evolution 201 (Thermo Fisher Scientific, Waltham, MA, USA) was used to characterize the optical transmission. The UV/Vis transmission spectra of FDM-printed PS (1100, 500 and 100 µm thickness) and commercial PS plates (1100 µm) were measured over a range of 250–750 nm.

### 2.7. Differential Scanning Calorimetry

The instrument DSC 204 *F1 Phoenix* (NETZSCH-Gerätebau GmbH, Selb, Germany) was used to investigate the glass transition temperature *T_g_* of FDM-printed PS samples by differential scanning calorimetry (DSC). Samples were heated from room temperature to 200 °C at a heating rate of 10 K/min.

### 2.8. Cell Culture

Mouse L-929 fibroblasts were grown in RPMI-1640 medium (supplemented with 10% FBS) at 37 °C and 5% CO_2_. The cells were cultivated in 75 cm² flasks for 1 week until reaching approximately 95% confluence. As FDM-printed substrates show an inherent laminate structure at the terminating layer and a smoother bottom side which faced against the building platform while printing, the influence of the sample topology on the cell proliferation was investigated. Therefore, cell culture experiments were performed with two different sets of substrates, either seeding the cells onto the laminate structured side facing upwards (PS rough) or onto the rotated substrates, which exhibit a smoother bottom side (PS smooth). FDM-printed substrate plates (0.5 mm thickness, 12 mm) were mounted into 24 well plates with Replisil 22 N and cured for 30 min. Afterwards, the substrates were sterilized for 30 min using 500 µL of 70% EtOH per well. After washing two times for 5 min with DPBS (−/−), the substrates were covered with 500 µL of RPMI for preconditioning at standard cell culture conditions for 2 h. Afterwards, the medium was replaced with 1 mL of ddH_2_O per well and the plates were stored at 4 °C overnight. To compare the printed PS samples to commercial TCPS as controls, wells of the same plates were preconditioned in the same manner. L-929 cells were seeded on the FDM-printed substrate or the commercial TCPS well plate and cultivated in 1 mL of RPMI-1640 medium per well with 10% FBS and 1% P/S for 24, 96 or 168 h in standard conditions. For the cultivation periods of 24 and 96 h, 1 × 10^4^ cells per well and for the cultivation period of 168 h, 2 × 10^3^ cells per well were seeded.

### 2.9. Live/Dead Staining

The viability of L-929 cells grown on 3D printed PS substrates and TCPS well plates were analysed after 24, 96 and 168 h. For this purpose, three independent experiments comparing the different samples (PS smooth, PS rough, control and dead control on TCPS) were carried out in triplicates. For the dead control, 500 µL of 0.1% Triton-X100 in DPBS (−/−) were added to the culture medium and incubated for 20 min at standard cell culture conditions. 

For the live/dead staining, the culture medium was removed and 500 µL of the staining solution containing 1 µM calcein-AM, 10 µM PI and 10 µg/mL Hoechst 33342 in DPBS (+/+) were added. After 30 min of incubation at 37 °C and 5% CO_2_, the cells were subjected to imaging using a confocal laser scanning microscope (LSM 980, Zeiss), equipped with the Zen software version 3.0 (Zeiss). For imaging, the staining solution was replaced with DPBS (+/+). The prepared dead control was used to set the laser power for the detection of the dead cells. For image analysis, the Fiji software (www.imagej.net/Fiji) was used [43]. The number of dead cells and the total cell number was determined. 

Analysis of the data and creation of the graphs was performed using the GraphPad Prism Software (version 5.01 for Windows, GraphPad Software, San Diego, CA, USA, www.graphpad.com). For the figure creation, the Inkscape software (version 1.0.2-2 for Windows, www.inkscape.org) was used.

## 3. Results and Discussion

### 3.1. Extrusion of PS-Filament for FDM Printing

**Figure 1:**
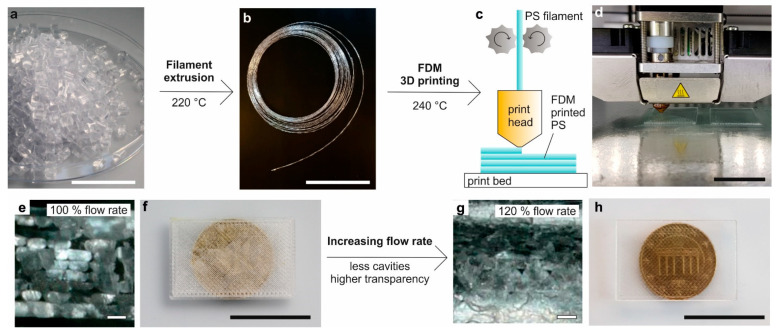
Principle of FDM printing of transparent PS. (**a**) From commercially available PS granules (scale bar 2 cm), a continuous, windable filament (**b**) with a mean diameter of 2.6 mm was prepared by continuous extrusion using a twin-screw extruder (scale bar 20 cm). (**c**) The filament was used to 3D print transparent PS components on a commercially available FDM printer. (**d**) FDM printing of a planar transparent PS substrate (scale bar 2 cm). (**e**) Cross-section of a PS substrate that was FDM printed with a material extrusion flow rate of 100%. The strands are not completely fused together resulting in air inclusions (scale bar 200 µm). (**f**) A 1.1 mm-thick PS substrate that was FDM printed with 100% flow rate. The air inclusions decrease the transparency (scale bar 2 cm). (**g**) Increasing the flow rate to 120% yields a higher density and substantially reduced the amount of air inclusions (scale bar 200 µm). (**h**) A 1.1 mm-thick PS substrate that was FDM printed with a flow rate of 120%. Due to the decreased amount of air inclusions the transparency is significantly increased (scale bar 2 cm).

To study FDM printing of PS for the manufacturing of transparent microfluidic devices on a laboratory scale, we first prepared a continuous PS filament by means of extrusion using a twin-screw extruder equipped with a 2.85 mm diameter nozzle. To prevent the diameter from becoming too big due to swelling, we pulled the extruded filament with a constant speed through a water-cooling bath. The resulting filament had a mean diameter of 2.6 ± 0.2 mm and could be wound up (Figure 1b). 

### 3.2. FDM of PS Microfluidic Devices

The PS filament was 3D printed using commercially available FDM printer (Figure 1c,d) to fabricate 3D PS parts. We evaluated the influence of the material flow rate on the transparency of the final PS components. Printing with a material flow rate of 100% yields PS samples with a low transparency (Figure 1e). An analysis of the part cross-section showed that the strands did not fuse together properly due to a lack of material and therefore generating small air inclusions between the deposited PS strands. Increasing the flow rate and therefore the amount of deposited material to 120% increases the transparency substantially. The cross-section analysis shows a denser PS part with lower amount of air pockets (Figure 1f). The material flow rate of 120% was used for all further prints.

**Figure 2:**
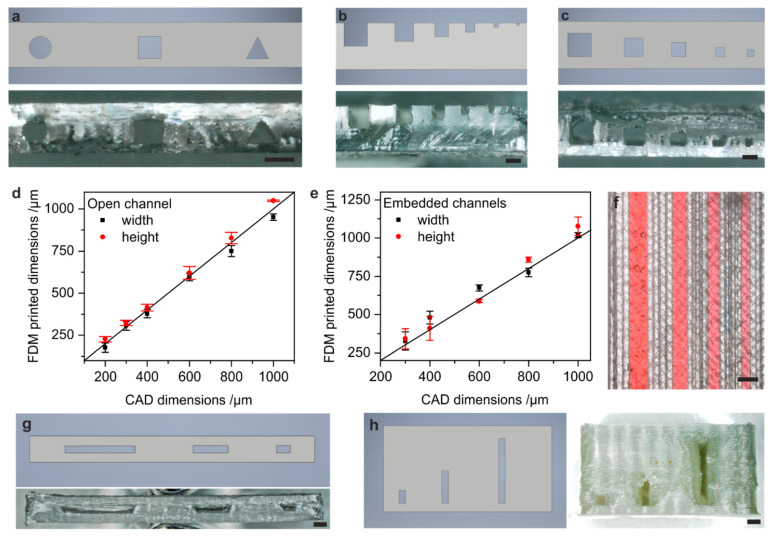
Accuracy and resolution of FDM-printed microfluidic channels in PS. (**a**) Cross-section of CAD model and FDM-printed PS, respectively, showing the possibility to print spherical, square and triangular channel cross-section with 600 µm features in high quality (scale bar 600 µm). (**b**) Cross section of CAD model and FDM-printed PS, respectively, showing open test channels with channel widths and heights of 1000, 800, 600, 400, 300 and 200 µm (scale bar 600 µm). (**c**) Cross section of CAD model and FDM-printed PS, respectively, showing embedded square test channels with channel widths and heights of 1000, 800, 600, 400 and 300 µm (scale bar 600 µm). (**d**) Comparison of the actual widths of FDM-printed open and embedded channels, shown in (**b**,**c**), over the original CAD dimension. (**e**) Comparison of the actual widths and heights of FDM-printed embedded microchannels shown in (**c**) over the original CAD dimensions. (**f**) The embedded microchannels shown in (**c**) were filled with dyed water to show full functionality (scale bar 1 mm). (**g**) Cross section of CAD model and FDM-printed PS, respectively, showing embedded test channels with lower aspect ratios of 0.5, 0.2 and 0.1 (scale bar 1 mm). The channel height was kept constant at 600 µm and the channel width was 1.2, 3 and 6 mm. (**h**) Cross section of CAD model and FDM-printed PS, respectively, showing embedded test channels with higher aspect ratios of 2, 5 and 10 (scale bar 1 mm). The channel width was kept constant at 600 µm and channel height was 1.2, 3 and 6 mm.

To evaluate the printability of microfluidic channels in PS we analysed the influence of the channel geometries on the printing accuracy. For this, we printed and evaluated straight test channels with varying channel cross-section geometries (0.6 mm width, spherical, square and triangular). Figure 2a shows the FDM-printed cross-section of the different channel geometries as well as the respective CAD model. It can be seen that all cross-sections can be successfully printed at high accuracy with minimal sagging. For all further prints, we chose to print with the square channel cross-sections.

To evaluate the accuracy and achievable resolution of the FDM-printed channels, we printed various open and embedded square test channels with channel widths and heights of 1000, 800, 600, 400, 300 and 200 µm, respectively. The cross-sections of the printed test channels as well as their respective CAD models are shown in Figure 2b,c. The actual dimensions of the open and embedded channels were measured and compared to their original CAD dimensions. Open channels can be printed with high accuracy down to a 200 µm channel width and height following a linear trend matching the original CAD dimensions (Figure 2d). Embedded channels can be printed with high accuracy down to channel widths and heights of 300 µm, following a similar linear trend as observed for the open channels (Figure 2e). Due to minor sagging, embedded channels with 200 µm widths and heights showed partial clogging and could not be printed reproducibly. The actual dimensions of embedded channels bigger than 300 µm were not influenced by sagging. The embedded test channels were filled with coloured water to demonstrate the channel function. As can be seen, no leakage was observed (Figure 2f). We further analysed achievable channel aspect ratios that can be fabricated by FDM printing of PS. To show aspect ratios smaller than 1, we printed straight test channels with a constant channel height of 600 µm and channel widths of 1.2 mm (aspect ratio 0.5), 3 mm (aspect ratio 0.2) and 6 mm (aspect ratio 0.1). A cross section of the FDM-printed channels is shown in Figure 2g. It was found that channel aspect ratios down to 0.2 could be successfully printed. Lower aspect ratios resulted in the clogging of the channels due to sagging of the bridging layers. We additionally analysed printing of microfluidic channels with aspect ratios higher than 1 by printing straight test channels with a constant channel width of 600 µm and a channel height of 1.2 (aspect ratio 2), 3 (aspect ratio 5) and 6 mm (aspect ratio 10), as can be seen in Figure 2h. High aspect ratio channels up to an aspect ratio of 10 were successfully printed in PS. High aspect ratio channels are more easily available using FDM printing than low aspect ratio channels, since the sagging problem has less influence on the channel geometry.

We printed test channels with layer thicknesses of 100 and 50 µm, respectively, and found that the printing accuracy and achievable resolution were not affected by the printed layer thickness. Since printing with a layer thickness of 50 µm at least doubled the printing duration, all further prints were prepared using a layer thickness of 100 µm.

**Figure 3:**
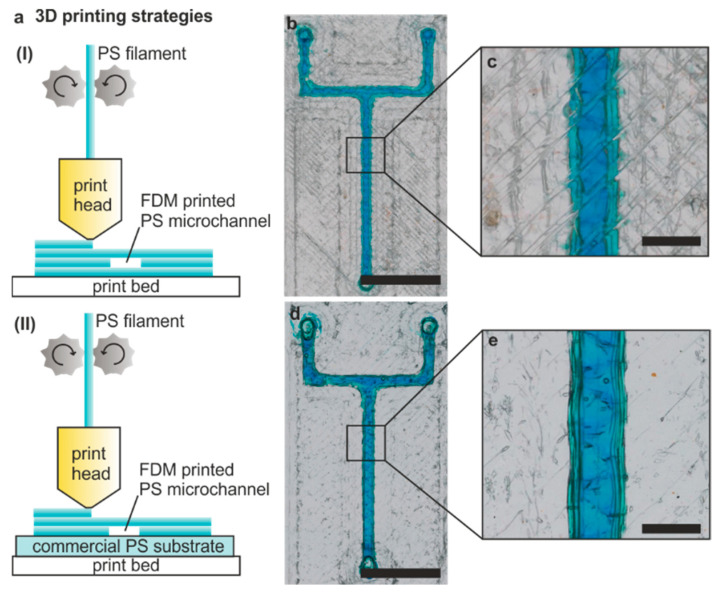
Three-dimensional printing strategies to improve microfluidic channel transparency. Two different strategies were used to 3D print microfluidic devices in PS: (**a**) printing directly onto the print bed (strategy I) and printing on top of a commercial PS foil (strategy II). (**b**,**c**) An exemplary Y-channel with a square cross-section channel of 600 µm widths and height printed using strategy I (scale bar 5 mm). The inherent layer structure reduces the transparency of the microchannels. (**d**,**e**) The same Y-chip as in (**b**) but printed with printing strategy II, resulting in a microfluidic channel with higher transparency in the region of interest (scale bar 600 µm).

To improve the transparency of the PS microchannels in the region of interest, we investigated two different printing strategies (Figure 3a). For strategy I, the chip was printed directly onto the print bed with a single bottom layer (0.1 mm), acting as a lid, beneath the actual channel structure. After filling the channel with coloured water, the deposited strands from the FDM print can be seen within the bottom layer, reducing the transparency of the microfluidic channel slightly (Figure 3b,c). 

To increase the optical transparency, we further evaluated FDM printing directly onto a commercial PS substrate (strategy II, Figure 3a). Upon deposition, the PS strands bond to the substrate and thus form embedded, sealed microchannels with the substrate acting as a lid. Despite the increase in z-axis height, which was adjusted prior to printing, no additional modification to the printing parameters was needed. Due to the lack of layers, microchannels printed with strategy II show an improved transparency in the region of interest (Figure 3d,e).

**Figure 4:**
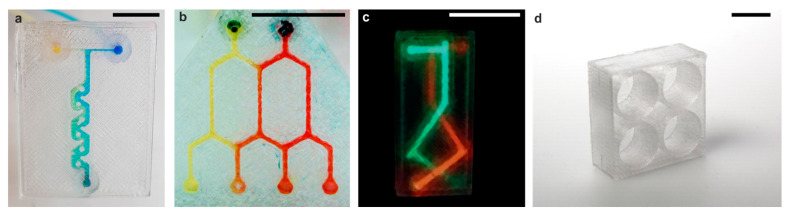
Applications of FDM printing of transparent PS. (**a**) A 2.5-dimensional microfluidic tesla-mixer with a square channel cross-section of 600 µm width and height. A blue and a yellow dye were mixed along the cascade. (**b**) A 2.5D mixer cascade with a square channel cross-section of 600 µm width and height. The mixing of a yellow and red dye demonstrates the creation of mixing gradients. (**c**) An FDM-printed microfluidic structure with two intertwining 3D microchannels of 600 µm width and height filled with fluorescent dyes to demonstrate the three-dimensionality. (**d**) An exemplary FDM-printed customized well-plate (scale bars 10 mm).

Several exemplary PS microfluidic chips showing high transparency were printed using strategy I, to highlight the versatility of FDM printing of transparent PS regarding rapid prototyping of microfluidic chips. The microfluidic chips shown in Figure 4 were printed using the square channel cross-section geometry with 600 µm channel widths and heights. We printed a Tesla-type micromixer as well as a cascade mixer, showing that complex and functional microfluidic components can be fabricated (Figure 4a,b). The printed chips showed good mixing behaviour. For the Tesla-type mixer (Figure 4a), we determined the mixing efficiency *c_m_* to be 0. 922 ± 0.008. The gradient mixer (Figure 4b) gave similar mixing efficiencies *c_m_* of 0.977 ± 0.009, 0.94 ± 0.02, 0.910 ± 0.008 and 0.965 ± 0.003 (from left to right). A microfluidic structure such as the Tesla-mixer can be produced in less than 1 h. A big advantage of FDM printing for rapid prototyping is that it allows the fabrication of actual 3D channels in contrast to commonly used replication techniques, which only allow 2.5-dimensional channels. Figure 4c shows an exemplary microfluidic structure containing two separate 3D channels. 

In addition, the FDM printing of transparent PS is not only limited to fabrication of microfluidic devices. As shown in Figure 4d, this process also enables the fabrication of bigger PS components such as customized well plates for life science applications. 

### 3.3. Surface Contact Angle

The surface properties of FDM-printed PS samples were characterised by measuring the static contact angle of water. Commercial PS samples were measured as a reference and the values were compared to the literature. Values of 84° ± 5° (4 measurements) were found for FDM-printed PS surfaces. The contact angle of commercial PS was found to be 85° ± 2° (4 measurements). These values are in accordance with values from the literature, namely static contact angles of 88° to 91° (e.g., cited by Kwok et al. [44] or by Ellison and Zisman [45]).

### 3.4. UV/Vis Spectroscopy and DSC Measurements

**Figure 5:**
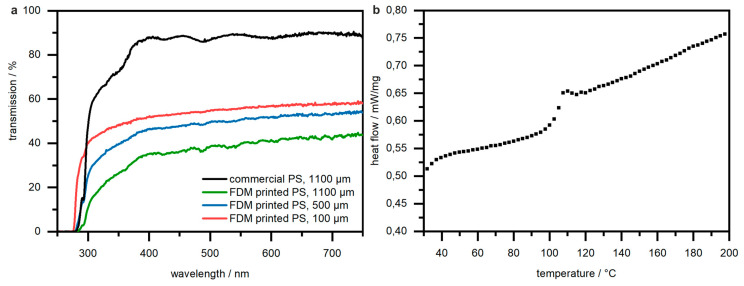
(**a**) UV/Vis transmission measurements on PS substrates with varying thickness (commercial PS for reference). Transmission values up to 58% were achieved with a layer thickness of 100 µm, whereas the maximum transmission was 44% for thicknesses up to 1100 µm. (**b**) DSC graph of FDM-printed PS. Samples were heated from 20 to 200 °C with a heating rate of 10 K/min. The glass transition of the sample can be seen with an onset at 100 °C.

One of the main benefits of using PS as a material for microfluidics is the high transparency. In order to demonstrate similar optical properties of FDM-printed PS and commercial PS, we characterized FDM-printed and commercial PS substrates by UV/Vis spectroscopy. The resulting spectra are shown in Figure 5. FDM-printed PS shows a lower UV/Vis transmission than commercial substrates due to the inherent microstructure of FDM-printed parts. The deposition of PS strands yields rough surfaces and air inclusions that scatter the light and reduce the transparency. With our optimized printing process, we achieve a transmission of >35% for 1.1 mm thick samples over a range of 750 to 400 nm. FDM-printed PS with a thickness of 100 µm shows a higher transmission of >51% over a range of 750 to 400 nm. Channel covers with these thicknesses can be created using this printing techniques, thus hinting that UV/vis spectroscopy is possible with a fully FDM-printed component. If higher transparency is required in a region of interest, printing strategy II, i.e., direct printing on top of a PS substrate, can be employed (Figure 3).

Furthermore, FDM-printed PS samples were analysed by DSC measurements to investigate the glass transition temperature *T_g_*. The resulting DSC graph is shown in Figure 5b. The *T_g_* of our FDM-printed PS samples was found to be 100 °C, which is in accordance with the literature [46]. These properties qualify FDM-printed PS to be used for applications such as polymerase chain reactions (PCR) on microfluidic chips, which require temperatures up to 95 °C during the denaturation step for their replication cycles [47].

### 3.5. Live/dead Staining

**Figure 6:**
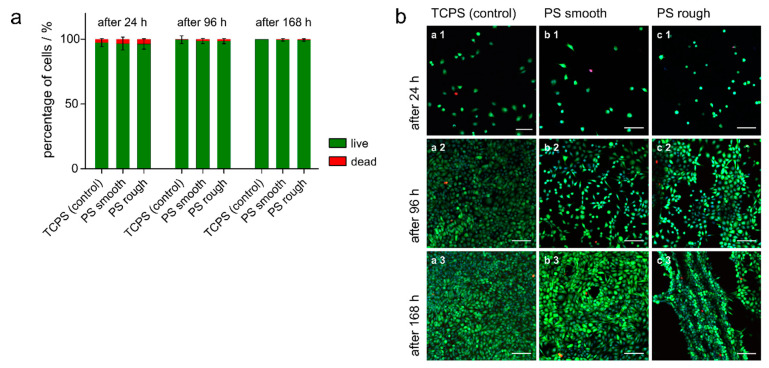
Results of the live/dead staining containing calcein-AM (green fluorescence, viable cells), PI (red fluorescence, dead cells) and Hoechst (blue fluorescence, all cells). Three independent experiments were carried out in triplicate. Four images per sample were taken (36 per condition per time point). (**a**) Percentages of live (green) and dead (red) L-929 cells grown on TCPS and FDM-printed PS substrates (smooth and rough surfaces) after 24 (left), 96 (middle) and 168 h (right). (**b**) Representative confocal LSM images of L-929 cells cultured for 24 (1), 96 (2) and 168 h (3) on TCPS (a) and FDM-printed PS substrates (b: smooth surfaces, c: rough surfaces) after a live/dead staining (scale bar 100 µm).

**Figure 7:**
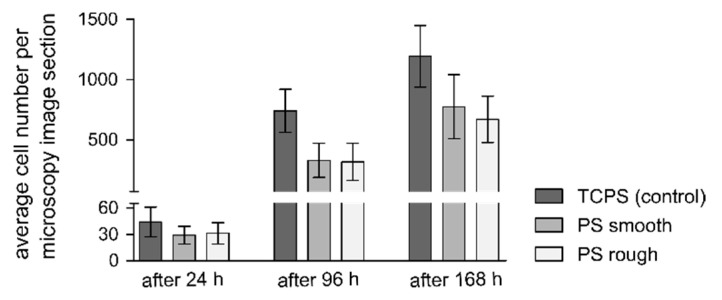
Average number of L-929 cells per image section after culture for 24 h (**left**), 96 h (**middle**) and 168 h (**right**) on TCPS (dark grey) and FDM-printed PS substrates (light grey: smooth surfaces, white: rough surfaces). The cell number was assessed by the counting of Hoechst-stained cells. For the cultivation periods of 24 and 96 h, 1 × 10^4^ cells per well and for the cultivation period of 168 h, 2 × 10^3^ cells per well were seeded. Three independent experiments were carried out in triplicate. Four images per sample were taken (36 per condition per time point). Error bars represent the standard deviation.

The viability of L-929 cells on FDM-printed PS substrates and TCPS well plates was compared after 24, 96 and 168 h using live/dead staining with calcein-AM, PI and Hoechst. In all conditions at the three time points, the viability of the cells on the printed substrates was comparable to the cells grown on TCPS (Figure 6a). Representative micrographs of the stained cells on the substrates at the different time points are shown in Figure 6b. Only a few dead cells (red fluorescence) can be seen and an increase in the number of cells per microscopic view field over time, indicating cell proliferation, was observable in all conditions. However, cells grew to higher numbers on TCPS compared to the printed PS substrates (Figure 7). On the rough surfaces, the printed grid guided the cells, resulting in cell growth mainly along and inside the grooves (Figure 6b,c3). These results indicate that the FDM-printed PS is not cytotoxic and enabled cell adhesion and proliferation. When assessing the results of the different proliferation rates observed in the live/dead staining, it must be considered that TCPS, which is a gold standard cell culture material and is used here as a control, is plasma-treated, which was shown to enhance cell growth and spreading in comparison to native PS [48]. The differences in cell growth on FDM-printed PS and TCPS is likely caused by the differences in the surface properties of the materials. The observed high cell viability during seven days of culture makes the FDM-printed PS a promising material for future cell culture applications.

## 4. Summary and Conclusions

In this work, we demonstrated a novel route to prepare prototypes of microfluidic chips in PS on the laboratory scale. Conventional prototyping of microfluidics to date is mostly conducted using PDMS, which causes a shift of material properties when migrating from small-scale to industry-scale production. Our process overcomes this problem, as it allows for fabricating microfluidic chips in PS using FDM printing technology. Commercially available PS granulate was used to prepare FDM-printable filament by continuous extrusion and printed using a commercial FDM printer. The printing settings were adjusted to improve transparency and allow printing of microfluidic structures with various channel geometries and dimensions down to 200 µm. Features smaller than 200 µm are so far not accessible due to the limited resolution of the FDM process. The resolution could be potentially improved using a smaller nozzle diameter of, e.g., 0.25 mm instead of 0.4 mm [25]. By printing on a PS substrate, we showed that the transparency in the region of interest can be significantly improved. To highlight the versatility of PS FDM printing, several exemplary geometries were printed, ranging from complex microfluidic structures such as Tesla-mixers and mixer cascades to customizable well plates. The transparency of FDM-printed PS was evaluated using UV/Vis measurements. By optimizing the printing process, we achieved a transmission higher than 50% over a range of 400–750 nm. We also conducted cell culture experiments indicating that FDM-printed PS is not cytotoxic and enabled cell adhesion and proliferation over a time period of 168 h, making it only slightly worse than TCPS, the reference gold standard. We could show that our printed PS has a Tg of 100 °C, thus enabling bioanalytical on-chip applications like PCR on chip, where temperatures up to 95 °C are needed [47]. We believe that this method for rapid prototyping of PS on the laboratory scale using 3D printing will be an important step to facilitate and accelerate the development of novel mass-market manufacturable microfluidic chips in PS.

## Data Availability

Not applicable.
